# Study protocol for a factorial-randomized controlled trial evaluating the implementation, costs, effectiveness, and sustainment of digital therapeutics for substance use disorder in primary care (DIGITS Trial)

**DOI:** 10.1186/s13012-022-01258-9

**Published:** 2023-02-01

**Authors:** Joseph E. Glass, Caitlin N. Dorsey, Tara Beatty, Jennifer F. Bobb, Edwin S. Wong, Lorella Palazzo, Deborah King, Jessica Mogk, Kelsey Stefanik-Guizlo, Abisola Idu, Dustin Key, John C. Fortney, Rosemarie Thomas, Angela Garza McWethy, Ryan M. Caldeiro, Katharine A. Bradley

**Affiliations:** 1 Kaiser Permanente Washington Health Research Institute, 1730 Minor Ave, Seattle, WA 98101 USA; 2 Department of Health Systems and Population Health, School of Public Health, University of Washington, Box 351621, 3980 15th Ave. NE, Fourth Floor, Seattle, WA 98195 USA; 3 Department of Veterans Affairs, Health Services Research and Development, Center of Innovation, 1660 S Columbian Way, WA 98108 Seattle, USA; 4 Department of Psychiatry and Behavioral Sciences, School of Medicine, University of Washington, Box 356560, 1959 NE Pacific Street, Seattle, WA 98195 USA; 5 Kaiser Permanente Washington Mental Health & Wellness Services, 1200 SW 27th St, Renton, WA 98057 USA

**Keywords:** Substance use disorders, Opioid use disorders, mHealth, Primary care, Implementation science, Factorial trial

## Abstract

**Background:**

Experts recommend that treatment for substance use disorder (SUD) be integrated into primary care. The Digital Therapeutics for Opioids and Other SUD (DIGITS) Trial tests strategies for implementing reSET® and reSET-O®, which are prescription digital therapeutics for SUD and opioid use disorder, respectively, that include the community reinforcement approach, contingency management, and fluency training to reinforce concept mastery. This purpose of this trial is to test whether two implementation strategies improve implementation success (Aim 1) and achieve better population-level cost effectiveness (Aim 2) over a standard implementation approach.

**Methods/Design:**

The DIGITS Trial is a hybrid type III cluster-randomized trial. It examines outcomes of implementation strategies, rather than studying clinical outcomes of a digital therapeutic. It includes 22 primary care clinics from a healthcare system in Washington State and patients with unhealthy substance use who visit clinics during an active implementation period (up to one year). Primary care clinics implemented reSET and reSET-O using a multifaceted implementation strategy previously used by clinical leaders to roll-out smartphone apps (“standard implementation” including discrete strategies such as clinician training, electronic health record tools). Clinics were randomized as 21 sites in a 2x2 factorial design to receive up to two added implementation strategies: (1) practice facilitation, and/or (2) health coaching. Outcome data are derived from electronic health records and logs of digital therapeutic usage. Aim 1’s primary outcomes include reach of the digital therapeutics to patients and fidelity of patients’ use of the digital therapeutics to clinical recommendations. Substance use and engagement in SUD care are additional outcomes. In Aim 2, population-level cost effectiveness analysis will inform the economic benefit of the implementation strategies compared to standard implementation. Implementation is monitored using formative evaluation, and sustainment will be studied for up to one year using qualitative and quantitative research methods.

**Discussion:**

The DIGITS Trial uses an experimental design to test whether implementation strategies increase and improve the delivery of digital therapeutics for SUDs when embedded in a large healthcare system. It will provide data on the potential benefits and cost-effectiveness of alternative implementation strategies. ClinicalTrials.gov Identifier: NCT05160233 (Submitted 12/3/2021). https://clinicaltrials.gov/ct2/show/NCT05160233

**Supplementary Information:**

The online version contains supplementary material available at 10.1186/s13012-022-01258-9.

## Contributions to the Literature

- In this randomized controlled trial, the FDA-authorized reSET and reSET-O digital therapeutics are being implemented in primary care clinics
- The study evaluates the extent to which health coaching and practice facilitation each improve the implementation over a standard implementation strategy
- Sustainment will be studied, and formative evaluation is used to monitor adaptations and optimize the success of implementation
- Findings regarding the population-level cost effectiveness of implementation strategies will further provide information to decision makers about the financial implications of this study’s implementation strategies

## Background

Substance use disorders (SUD) are prevalent, undertreated, and costly to society [1–5]. Approximately 265 people a day died from drug overdose in the United States in 2021 [6]. However, just 8-14% of people with past-year SUD receive treatment [1, 2]. Most individuals with SUD prefer to receive treatment in primary care [7], and it is believed that providing SUD care in primary care would be less stigmatizing [8]. Consequently, to increase access to SUD treatment, the National Academies of Medicine, Science, and Engineering and others have called for increased integration of substance use care in primary care [9–15].

New ways to address SUD in primary care are needed. Health systems have taken steps to prevent and treat SUD in primary care through screening, brief intervention, referral to specialty care, and medication treatments that can be prescribed by primary care providers (PCPs) [13–21]. However, health systems have been unable to effectively provide treatment to the high volume of patients with SUD who visit primary care [22, 23]. Implementation of SUD treatments is often hindered by feasibility problems, lack of capacity, and discomfort in treating SUD in primary care [24–26]. For instance, traditional treatments such as cognitive behavioral therapy and contingency management are among the most proven psychosocial treatments for SUD [27–31], but may require extensive resources to implement and deliver [32, 33]. Buprenorphine, a life-saving treatment for opioid use disorder (OUD), can be prescribed in office-based settings, but most do not receive this medication [34–36]. There is a clear need to identify treatments that are both effective and feasible to implement in primary care.

The adoption of digital therapeutics in primary care is potentially one way to increase access to evidence-based treatments. Several digital therapeutics for SUD are supported by evidence for their efficacy or effectiveness [37–39]. Digital therapeutics may deliver intervention content such as assessments, treatment modules, and normed feedback to patients via websites or smartphone apps, often under the guidance of a clinician [40]. The use of digital therapeutics could potentially help overcome common barriers in primary care, while providing access to an effective treatment [41, 42]. For instance, studies have shown that digital therapeutics for SUD can produce beneficial effects while reducing the amount of time that clinicians need to spend with patients [39, 43], they are acceptable to patients [44, 45], they may improve clinical outcomes when delivered in real-world care [46–49], and they can be effective when added to usual care approaches that lack an evidence-base [39, 43].

At least two logistical challenges must be addressed in implementation research to determine how to achieve a far-reaching, sustainable implementation of digital therapeutics in primary care. First, health systems must solve barriers to getting clinicians to offer these treatments to patients. For instance, clinicians encounter difficulty with executing novel workflow processes [50–54], such as creating login accounts for patients and “teaching” them to use apps [40, 55, 56], often impacting adoption of these treatments [52, 57, 58]. A second challenge is that patients often need human support to effectively engage in digital therapeutics [53, 54, 59–62]. Successful implementations must provide support to patients to help them engage in use of apps, without overburdening primary care teams [51, 53, 54, 59, 63]. Given the known time constraints and competing demands in primary care [25, 64], teams may find it infeasible to offer adequate support for engagement in digital therapeutics. It is unknown whether clinicians in primary care can add additional tasks to their already demanding workload to encourage digital therapeutic engagement, or if they alternatively need dedicated staff to ensure engagement.

### The DIGITS Trial

The Digital Therapeutics for Opioids and other SUDs trial (DIGITS Trial) seeks to identify how to best implement digital therapeutics for SUDs in primary care. The clinical intervention includes two 12-week, smartphone-based prescription digital therapeutics, reSET® and reSET-O® made by Pear Therapeutics, which have been authorized by the United States Food and Drug Administration (FDA) for the treatment of SUD and OUD, respectively. reSET and reSET-O are commercial versions of a computerized cognitive-behavioral treatment, the Therapeutic Educational System, which was shown to improve patient outcomes in four RCTs [39, 43, 65–67]. However, all prior RCTs were conducted in specialty addiction treatment settings, not in primary care.

The DIGITS Trial seeks to evaluate whether practice facilitation and health coaching can improve digital therapeutic deployment in primary care beyond a standard implementation strategy. The standard implementation strategy, which serves as a comparator, is based on a multifaceted implementation strategy previously used by clinical leaders at the participating healthcare system to implement app-based treatments for depression and anxiety. It includes discrete strategies such as clinician training and electronic health record (EHR) tools.

*Practice facilitation* is designed to overcome workflow challenges by supporting clinicians to tailor implementation to their local context [68–70]. Facilitation methods have been used to implement addiction interventions and experts describe it as one of the most successful implementation strategies [58, 71–75]. It is both a process and a set of strategies designed to build relationships, identify and overcome barriers, and help the clinical team with implementation. Meta-analysis has demonstrated that facilitation increases the odds of evidence-based primary care [76].

*Health coaching* employs a centralized mid-level provider, namely, a medical assistant (MA), to coach patients to engage in the digital therapeutic while reducing burden on primary care teams. The health coach encourages patient engagement and use of the apps, reinforces learning and skill practice, and encourages completion of healthcare visits with the primary care team. The strategy’s conceptual targets are informed by the literature on patient-mediated implementation strategies, such that health coaching is designed to inform and educate patients, activate them in healthcare, and promote collaboration between patients and healthcare teams [77]. Coaching is an effective strategy for engaging patients in digital therapeutics [53, 54, 59–61].

The DIGITS Trial is a parallel group, factorial, cluster-randomized trial. Cluster-randomization is at the clinic level because the implementation strategies were assigned to primary care clinics. The strong evidence for reSET and reSET-O from specialty care settings provides sufficient inferential evidence to conduct a trial in primary care that uses a hybrid effectiveness-implementation design with a main focus of evaluating implementation outcomes and a secondary focus of evaluating population-level effectiveness and cost-effectiveness of implementation strategies (“hybrid type 3”) [78].

#### Specific Aims

The first aim is to estimate the effect of practice facilitation and health coaching in increasing the reach and fidelity [79, 80] of digital therapeutics. We hypothesize significantly higher reach among clinics randomized to practice facilitation (hypothesis 1) and significantly higher fidelity among clinics randomized to health coaching (hypothesis 2), compared to clinics that did not implement with these respective strategies.

The second aim is to compare the population-level cost-effectiveness (PLCEA) [81] of the implementation strategies in improving reach, fidelity, and substance use. This analysis will inform the economic value of the additional implementation strategies relative to the standard implementation strategy. PLCEA methods consider that real-world implementations of an evidence-based practice may yield different effectiveness or cost-effectiveness results than tightly controlled RCTs of the same intervention.

Other study objectives are to: 1) conduct a formative evaluation to provide feedback to the healthcare system, monitor implementation fidelity, and record adaptations, 2) evaluate additional secondary and other outcome measures, including sustainment of the implementation, to provide a comprehensive assessment of implementation success, and 3) evaluate patient-level moderators of reach.

## Methods / Design

### Setting

The trial is conducted in primary care clinics of Kaiser Permanente Washington (KPWA), a health insurance coverage and healthcare system that serves a population of privately insured patients and those insured by Medicaid and Medicare. Additional file 1 contains details about the structure of primary care teams and SUD services available. KPWA provides care in urban, rural, and suburban communities. Patient populations of the primary care clinics vary in diversity; clinics range from 13-53% Black, Indigenous, or Persons of Color (includes Black/African American, American Indian/Alaskan Native, Asian, Native Hawaiian/Pacific Islander, Hispanic or Latino). Annually, approximately 340,000 patients attend healthcare visits.

### Overview of Trial Design

**Fig. 1:**
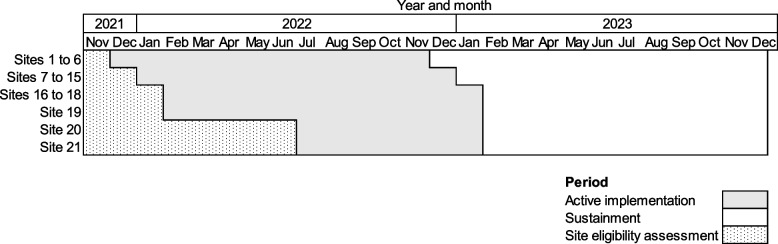
Timing of active implementation and sustainment periods at the study sites

Primary care clinics are eligible if they have ≥1 clinician trained in reSET and reSET-O and had not previously piloted these digital therapeutics. To maximize the number of study clinics, clinics could become eligible if they met criteria from 12/9/2021 through 8/11/2022 (Fig. 1). Each clinic has an active implementation period of a minimum of 6 months to a maximum of 12 months, starting on the clinic’s randomization date. Table 1 displays a chart of study eligibility assessment, allocation to study arms, and assessments periods following SPIRIT guidelines [82].

**Table 1:** Timing of the DIGITS Trial’s assessment schedules, following SPIRIT 2013 guidelines (Standard Protocol Items: Recommendations for Interventional Trials)

	Enrollment	Allocation	Post-allocation								Close-out
TIMEPOINT	-*t*_1_	0	*t*_1_	*t*_2_	*t*_3_	*t*_4_	*t*_5_	*t*_6_	*t*_7_	*t*_8_	*t*_x_
ENROLLMENT:											
Clinic eligibility assessment	X										
Allocation to study arm^1^		X									
PERIODS:											
Active implementation			X	X	X	X					
Sustainment							X	X	X	X	
INTERVENTIONS:											
reSET & reSET-O availability			X	X	X	X	X	X	X	X	
Standard implementation			X	X	X	X					
Practice facilitation			X	X	X	X					
Health coaching			X	X	X	X					
ASSESSMENTS:											
Covariates^2^	X	X	X	X	X	X	X	X	X	X	
Implementation costs	X	X	X	X	X	X					
Reach^3^ & adoption			X	X	X	X					
Fidelity, engagement			X	X	X	X	X				
Substance use, abstinence from screening data			X	X	X	X	X	X	X	X	X
Substance use, abstinence from reSET & reSET-O data			X	X	X	X	X				
Sustainment							X	X	X	X	

*t*_1_ through *t*_8_ are discrete 3-month periods. Sites with shorter active implementation and sustainment periods have shorter assessment periods. For instance, the reach & adoption assessment period will be 7 months for a site with a 7-month active implementation period.

^1^While sites are allocated to arms upon randomization, an open cohort design is used to identify patients for the analysis. Patients who visit a site after randomization, or in the 2 weeks before randomization, are eligible for the analysis. This 2-week period before randomization allows for the possibility that clinicians will identify patients with recent visits who could benefit from the intervention. ^2^Patient covariates are assessed in the year prior to a patient’s qualifying visit. ^3^For each patient, we will identify whether they were reached from the day of their first qualifying visit through the end of the active implementation period plus a 2 week “grace” period. The grace period allows patients who have their first qualifying visit at the end of active implementation at least 2 weeks to be reached.

Each clinic’s implementation approach is assigned using a 2x2 factorial design *(*see *Randomization Procedures)*. This allows this study to estimate the main effects of the implementation strategies, compare their effectiveness, and evaluate their interactions. This design yields four distinct implementation approaches (Table 2).

**Table 2:** Random assignment of primary care clinics to two implementation strategies resulting in four implementation approaches (2x2 factorial design)

Implementation Strategy	Approach 1: Standard Implementation Only	Approach 2: Standard Implementation + Practice Facilitation	Approach 3: Standard Implementation + Health Coach	Approach 4 Standard Implementation + Both
Standard Implementation	YES	YES	YES	YES
Practice Facilitation	NO	YES	NO	YES
Health Coach	NO	NO	YES	YES

The study obtains all quantitative data for sample identification and outcome measurement from secondary data. The study is not proactively recruiting participants into the analytic sample; rather, outcomes will be analyzed among all patients who screen positive for unhealthy substance use in primary care, allowing for unbiased assessment of reach into the target population.

#### Ethical Considerations

The Kaiser Permanente Washington Institutional Review Board granted ethical approvals including providing waivers of consent and HIPAA authorization.

### Data Sources

#### Quantitative Data Sources

Data for the study are drawn from multiple sources, including: (1) EHR data, (2) health plan insurance claims, and (3) reSET and reSET-O usage and patient self-report files [46, 83]. Additional data for the economic evaluation are described later (see *Economic Costs*).

EHR and claims databases include information generated by patient visits inside and outside of the study healthcare system, respectively. Data domains include demographic characteristics, diagnosis codes, procedure codes, medications, visit type and location, and provider type. EHR databases additionally include behavioral health screening data.

### Randomization Procedures

**Fig 2:**
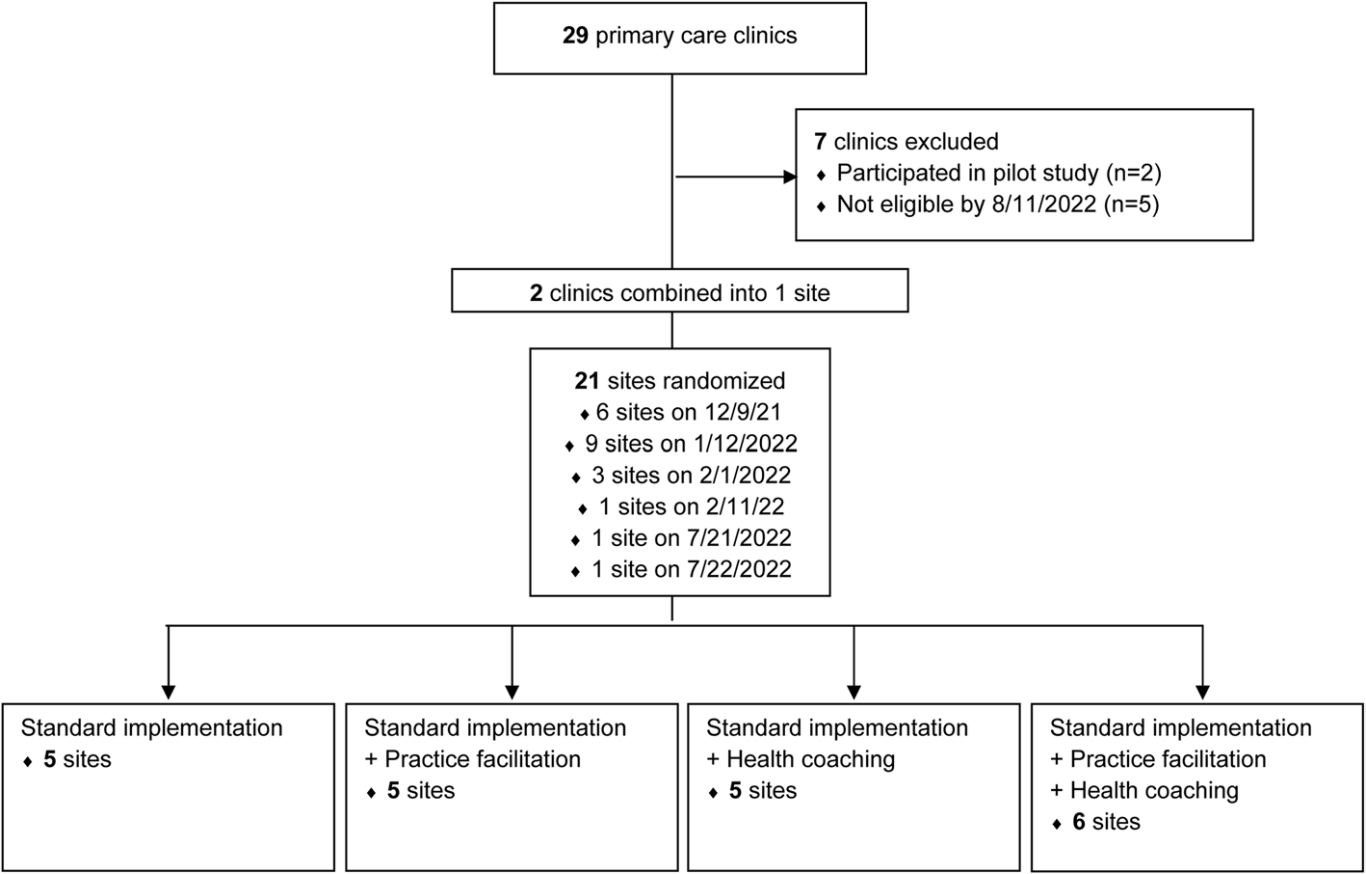
CONSORT diagram of site enrollment and allocation to study arms

Sites were randomized with equal probability to the four different implementation approaches by the study biostatistician using a computer-generated list of random numbers. Randomization employs permuted blocks of size 4 or 8 (randomly selected) to balance characteristics of clinics that become eligible for the study over time. The allocation sequence is concealed by the study biostatistician in a password-protected file until clinic eligibility is ascertained. A total of 22 clinics were randomized (Fig. 2). Two clinics were paired because of their geographical proximity and sharing of clinic staff, yielding 21 randomized sites.

### Conceptual Framework

This study uses the dynamic sustainability framework (DSF) to conceptualize the process of implementation and sustainment [84, 85]. In the DSF, three core constructs, the intervention, practice setting, and ecological system are constantly in flux. Changes are intrinsic in implementation efforts, thus implementation teams must seek to “maximize fit” by iteratively monitoring, recording, and adapting to changes over time to increase the likelihood of reach and sustainment [84]. A failure to maximize fit may lead to implementation failure and lack of sustainment. We chose an adaptive implementation strategy, practice facilitation, to attend to clinic-level barriers and facilitators that vary across clinics and over time [52, 86]. The DSF also guides our approach to formative evaluation and its continuous assessment of key DSF constructs (see *Formative Evaluation*).

#### Study Development and Piloting

From February to May 2021, researchers and healthcare system leaders partnered to conduct a quality improvement project to pilot the standard implementation strategy at two primary care clinics. Goals were to test and refine the clinical workflow for delivering reSET and reSET-O, and to evaluate and improve the acceptability and feasibility of the implementation strategy. Prior to piloting, the study required contracting with the vendor, healthcare system approval of the app’s use of monetary incentives, and an extensive information security risk evaluation. Initial workflow design was informed by two user-centered design studies conducted in partnership clinical leaders [51, 87]. After the quality improvement pilot, practice facilitation and health coaching were added to the pilot clinics. Learnings led to the modification of EHR tools, training materials and processes, procedures for virtual visits, and economic analysis and formative evaluation data collection materials. The health coaching protocol was modified to proactively inform patients about the digital therapeutics. We also conducted secondary data analyses of health system data to refine the statistical analysis plan and operationalization of study measures.

### Clinical Intervention

reSET and reSET-O can be prescribed to patients who attend in-person or virtual healthcare visits. Table 3 describes the general procedures used by clinicians to offer these treatments. Clinicians assess patient eligibility for being offered reSET and reSET-O based on the FDA label for each product. Instead of collecting written consent, text is generated by the EHR to notify patients that researchers are using medical records to study offering the apps. Background information about the selection of reSET and reSET-O for this study is included in Additional file 2.

**Table 3:** General procedures for using reSET and reSET-O in primary care

Initiate a patient onto reSET or reSET-O	Support for use of reSET or reSET-O
• Identify patients who may benefit from reSET or reSET-O^1^ (from warm hand-offs, referrals, scheduled appointments, population management registries) • Offer via shared decision making • Enter an electronic order for the treatment to facilitate a prescription • Provide instructions to the patient and an enrollment code for app activation • Follow-up with patients as indicated during the 12-week prescription	• Encourage completion of 4 modules per week • Monitor use of the app with the clinician dashboard • Identify barriers to engagement • Review content learned • Encourage skill practice • Consolidate what patients learn and discuss next steps

^1^Eligibility criteria for reSET and reSET-O can be briefly summarized as: aged 18 years or older, has a diagnosis of substance use disorder, has access to a tablet or smartphone with intervention access, primary language is English, and able to review the Terms of Service and Privacy Policy. reSET is indicated for patients with substance use disorder except sole alcohol use disorder or primary opioid use disorder. reSET-O is indicated for patients with opioid use disorder who are taking buprenorphine. The exact eligibility criteria are available on the Pear Therapeutics website.

### Implementation Strategies

#### Standard Implementation

Upon study launch, the healthcare system led a 3-hour, two-part live videoconference training on delivering reSET and reSET-O. The live training was offered three times to accommodate clinician schedules. Clinicians who missed these trainings could complete a “self-study” digitally recorded training. The healthcare system identified that integrated mental health specialists embedded in clinical teams would be the main digital therapeutic prescribers. Within three months after randomization, PCPs and nurses began to express interest in prescribing and thus were provided with self-study training materials.

Trainings cover research evidence for reSET and reSET-O, a product demo and evidence review by Pear Therapeutics staff, a description of the implementation toolkit and procedures for prescribing, a question & answer period, and a walkthrough of EHR documentation templates and tools. The implementation toolkit included: (1) a job aid with major steps of digital therapeutic delivery; (2) patient pamphlets that describe the digital therapeutics and steps for getting started; (3) scripts to help clinicians introduce the digital therapeutics; and (4) EHR tools including documentation templates, patient instructions, and a description of risks/benefits (e.g., app security and privacy information). These were adapted from a toolkit previously used by the healthcare system. New materials for this study included (5) an EHR order set to prescribe the digital therapeutics (“standing order”), and (6) a “huddle card” to guide local reSET and reSET-O prescribers in marketing the apps to primary care teams during standing meetings. All materials are continuously revised as needed and hosted on an intranet website. Healthcare system leaders offer continued support to clinicians on an as-needed basis. Health system leaders review monthly text-based performance reports of clinician and patient digital therapeutic usage data.

#### Practice Facilitation

In the context of a supportive relationship, facilitation is centered around four components of an implementation facilitation manual [88]:

1. **Bolster education:** Help the local clinic’s reSET and reSET-O prescriber learn how to market the therapeutic to patients and care teams and garner support for digital therapeutic use.
2. **Audit and provide feedback:** Share progress on measurable performance goals of reach and fidelity for self-assessment and individual performance ranking in comparison to other anonymized clinics to prompt change in practice.
3. **Support Plan-Do-Study-Act (PDSA) cycles**: Support the clinic’s reSET and reSET-O prescriber in designing small tests of change to increase intervention reach and fidelity. This involves reviews of audit and feedback data followed by goal setting, problem solving, and adjustment of activities (e.g., workflows).
4. **Engage others in change**: Invite additional implementation stakeholders (e.g., clinic leadership, PCPs, care team members) to participate in problem-solving and PDSA cycles.

Trained facilitators hold practice facilitation sessions over videoconference consistent with a virtual facilitation approach [88]. After randomization, the facilitator meets with the local reSET and reSET-O prescriber to establish a relationship, set expectations, and learn about the clinic context. The facilitator then meets with clinic leadership to set expectations and secure leadership endorsement of facilitation activities.

After the leadership meeting, the facilitator holds an implementation kickoff meeting with the local clinic reSET and reSET-O prescriber(s) (and health coach, if applicable), which is the first of 12 monthly facilitation meetings. Facilitators are available for ad-hoc support between meetings and keep a log of meeting length, attendance, and implementation activity completion. Meeting agendas are tailored in response to staff capacity and progress on the four facilitation components strategies. If the clinic’s prescriber leaves the organization, the facilitator will suspend monthly meetings until another staff is hired and/or trained, at which point meetings will resume with the new team member.

### Health Coaching

The health coach is a credentialed MA employed by the healthcare system (not by researchers). The healthcare system provides a supervision structure, credentialing, and oversight, and the research grant funds the position. The MA completed training and certification as a Certified Wellness Health Coach through Real Balance Global Wellness Services [89], paid for by the study. A study research interventionist developed the manualized coaching protocol, trained the health coach to follow the manual, and provides ongoing technical assistance in approximately weekly calls. The MA conducts visits from an office at a central location via telephone and electronic messages through the EHR patient portal. Before the first session, the health coach notifies patients that researchers are studying the impact of health coaching.

Health coaching includes approximately weekly coaching contact with enrolled patients and outreaches to those who are prescribed but do not initiate or engage with the therapeutic. The health coach discusses digital therapeutic lesson content with the patient and encourages them to engage in the content. The coaching protocol guides the MA to handle patient concerns (e.g., substance use, safety concerns, technical issues) directing clinical concerns to a clinician. The health coach documents contact with patients in the medical record. In addition, the health coach conducts proactive outreach by sending messages to potentially eligible patients via the patient portal to notify them about the availability of the digital therapeutics. To facilitate these activities, the health coach monitors an EHR-based population management workbench and the app vendor’s web-based clinical dashboard.

### Additional implementation strategy details

Additional file 3 follows reporting guidelines outlined by Proctor and colleagues to document the implementation strategies [90].

## Quantitative Evaluation

### Study Sample and Eligibility Criteria

Sites are the unit of analysis. The study uses an open-cohort design, identifying patients following randomization because the digital therapeutic is offered when patients have a clinical encounter. For the statistical analyses, patients will be attributed to the site where they had their first qualifying visit. Patient eligibility criteria for automatic inclusion are determined with EHR data. Patient inclusion criteria are (1) had a primary care visit in a participating clinic from 2 weeks before through the active implementation period (for primary outcome analyses) or sustainment period (for sustainment analyses), (2) screened positive for substance use on the day of the visit or in the prior year, and (3) adult 18 years of age or older at the time of the visit. Positive screens are indicated by patient self-report of daily cannabis use or any drug use in the past year on instruments described previously [91, 92]. Patients are excluded if they have previously requested to opt out of research studies.

### Outcome Measures

Table 4 outlines the study’s primary and secondary outcomes, other pre-specified outcomes, and other explanatory and sensitivity analysis measures. Outcomes are conceptualized via the RE-AIM framework (reach, effectiveness, adoption, implementation fidelity, maintenance/sustainment) and measured at the site-level [93, 94].

**Table 4:** Primary, secondary, and other outcomes in the DIGITS Trial^1^

**Primary Outcome Measures**	
*Reach*. Reach of the digital therapeutic to patients in the primary care clinic, measured as the proportion of patients who initiate the digital therapeutic, defined by instances in which patients open the app, enter the prescription code, and use a treatment module *Fidelity*. Fidelity of patients' use of the digital therapeutic to clinical recommendations, measured as the mean number of weeks during patients’ 12-week prescription in which patients use 4 app modules/week and have visited a clinician in the past 30 days^2,3^	
**Secondary Outcome Measures**	
*Engagement* (patient engagement in substance use care). Mean number of months in which patients make ≥1 visit for substance use disorder^2,3^	
*Economic costs*. Costs from the perspective of a health system and payer including implementation, direct intervention, operating, and other indirect healthcare costs. This measure will be used to calculate the population-level cost effectiveness of increasing reach, fidelity, and engagement.	
**Other Pre-Specified Outcome Measures**	
*Reach-2*. The proportion of patients prescribed the digital therapeutic	
*Fidelity-2*. Mean number of weeks in which patients use at least 1 module/week^3^	
*Substance use*. The proportion of patients who are reached and reduce their substance use^4^	
*Abstinence*. The proportion of patients who are reached and are abstinent from substances^4^	
*Sustainment*. The proportion of patients who are reached during the sustainment period.	
**Other Explanatory and Sensitivity Analysis Measures**	
*Adoption*. The proportion of healthcare provider prescribing the digital therapeutic, overall and by provider type	
*Adoption-2.* The mean number of months in which providers access clinician dashboards	
*Reach-3*. Proportion of patients who download and unlock the digital therapeutic	
*Fidelity-3*: Mean number of weeks in which the patients use at least 1 module/week^3^	
*Fidelity-4*: Mean number of weeks in which the patients use 4 modules per week but without the requirement that they visit a clinician^3^	
*Fidelity-5*: Mean number of modules completed over the 12-week prescription^3^	
*Substance use-2. The proportion of patients who are reached and reduce their substance use, as measured by self-report data collected by the digital therapeutic*	
*Abstinence-2.* The proportion of patients who are reached and are abstinent from substances, as measured by self-report data collected by the digital therapeutic	
*Abstinence-3. Abstinence verified by urine drug screens among patients prescribed the digital therapeutic for opioid use disorder, based on EHR data*	

EHR=electronic health record

^1^Primary, secondary, and other pre-specified outcome measures are described as registered on ClinicalTrials.Gov. The other explanatory and sensitivity analysis measures were not registered.

^2^Visits must indicate that the clinician coded an International Classification of Disease (ICD-10) substance use disorder diagnosis.

^3^Fidelity and engagement are measured while a prescription is active, even if the prescription starts before but ends after their clinic’s active implementation period is complete.

^4^Pre-specified substance use and abstinence are collected via self-report as part of routine annual screening for cannabis, alcohol, and other drug use [22]. The assessment period includes up to 18 months after each patient’s qualifying visit to help ensure a follow-up measure is collected. These will be analyzed among patients who become eligible before the last 3 months of active implementation (to help ensure the patients could complete the 12-week prescription before the outcomes were measured). Substance use will also be assessed separately by each type (i.e., drug-specific reductions in the frequency of cannabis use, illicit drug and prescription medications use, and alcohol use separately).

Reach and fidelity are primary outcomes. Reach is the site-level proportion of patients who initiate reSET or reSET-O. For this outcome to occur, a clinician must prescribe the digital therapeutic and the patient must complete at least one treatment module. Fidelity is the site-level mean number of weeks in which patients use the digital therapeutic as recommended. This includes completing a recommended 4 or more modules per week while under the care of a clinician [39, 95, 96].

Patient engagement in SUD care, a secondary outcome, is operationalized as the mean number of months in which patients make at least one visit for SUD in any setting. Engagement is conceptualized as an effectiveness outcome. Economic costs are another secondary outcome operationalized using PLCEA methodology (see *Economic Evaluation*).

Other pre-specified outcome measures include an additional measure of reach quantifying the proportion of patients who are prescribed reSET or reSET-O by a clinician, irrespective of whether a patient initiates its use. An additional fidelity measure will estimate the number of weeks in which patients complete at least one module per week. Two effectiveness outcomes include substance use and abstinence, measured as the proportion of patients who are reached and reduce their substance use, or who use no substances, respectively.

Sustainment will be operationalized as the proportion of patients who are reached (same definition as above) during the sustainment period of the study.

Exploratory measures will be analyzed to comprehensively assess implementation and effectiveness. One adoption measure is operationalized as the proportion of healthcare providers prescribing reSET or reSET-O, overall and by provider type. Another adoption measure is the mean number of months in which providers access clinician dashboards. An additional reach variable will indicate the proportion of patients who download and unlock the digital therapeutics. Two additional fidelity variables indicate the mean number of weeks in which patients use 4 modules per week regardless of whether they see a clinician, and the mean number of modules completed over the 12-week prescription. Additional substance use and abstinence measures will use self-report timeline follow-back data collected by the app every four days during the 12-week prescription [75]. We will also measure the proportion of patients who are reached by reSET-O and achieve abstinence from all substances, as evidenced by results of routine urine drug screens administered as part of clinical care (often administered among patients with OUD).

### Statistical Analysis

Following procedures for factorial trials, we will analyze main effects on primary and secondary outcomes by examining the mean responses at one factor level and at a contrasting factor level, collapsed across all levels of the other factors (e.g., practice facilitation versus no practice facilitation) [97–99].

#### Primary Outcome Analyses

Analyses will follow intent-to-treat principles, with site analyzed according to their assigned treatment group regardless of the amount of implementation strategy delivered. We will fit a linear regression model to estimate the main effect of each factor level (practice facilitation, health coach) [97, 99]. For the primary outcomes of reach and fidelity, we will apply linear regression to model the proportion of patients reached within a site and the site-specific mean number of weeks with fidelity, respectively. We will test hypotheses 1 and 2 by testing the appropriate contrast from the regression model and use the Holm procedure to control the familywise type 1 error rate of the two primary hypotheses at 0.05. In addition to the hypothesis above (see Specific Aims), we secondarily hypothesize that practice facilitation is superior to health coaching in increasing reach, and health coaching is superior to practice facilitation in increasing fidelity. We also will examine whether the two enhanced strategies together are superior to standard implementation by comparing clinics with both enhanced strategies to clinics with neither. We will also examine interaction effects between the two enhanced strategies.

#### Secondary and Other Pre-Specified Outcome Analyses

We hypothesize significantly higher engagement among sites randomized to practice facilitation or health coaching. Analyses will follow the same general modeling approach as the primary outcomes. Secondary outcome analyses will apply a traditional two-sided type 1 error rate of 0.05; considering issues of multiple comparisons, findings will be interpreted with caution. See *Economic Evaluation* for a description of cost analyses.

Additional pre-specified reach and fidelity measures and other outcomes will follow the same analytic procedures as the primary outcomes (e.g., linear regression of the site-level measures).

Sustainment analyses will describe reach over time during the sustainment period graphically by study arm. If reach is greater than 5% during sustainment (overall or in any study arm), we will conduct secondary analyses allowing the intervention effect to vary over time. Specifically, we will subdivide the sustainment period into discrete time intervals (e.g., 4-month windows) and include interaction terms with intervention group; repeated measures over time within a clinic will be accounted for using a mixed-effects model with site-specific random intercepts.

#### Patient-level moderators of reach and fidelity

We will conduct exploratory patient-level analyses stratified by sex and by SUD type when estimating reach, fidelity, abstinence, and substance use reductions, allowing us to provide data about implementation effectiveness in specific subgroups.

#### Sensitivity Analyses

We will conduct several sensitivity analyses to examine the robustness of trial results. If, due to random chance, factor levels are imbalanced in any baseline characteristics (e.g., site size), we will perform sensitivity analyses where we include these characteristics in the regression models. Following reporting guidelines [100, 101], we will examine whether the proportion of screen-positive patients (the measure defining the study population) differs across arms. To assess for identification bias [102, 103], we plan to conduct sensitivity analyses in which we consider alternative population denominators: all patients with a SUD diagnosis, and all patients with visits regardless of whether they screened positive for substance use.

#### Statistical Power

Minimal detectable differences for fixed 80% power were estimated based on 27 clinics (number of clinics available during the study pilot phase) and a two-sided type 1 error rate of 0.025 for the two primary outcomes of reach and fidelity (to control the familywise type 1 error at 0.05). We estimated that we will have >0.80 power to detect an increase of 2 percentage points in site-level reach among screen-positive patients in sites with versus without practice facilitation. We estimated that we will have >0.80 power to detect an increase in the site-level mean number of weeks fidelity of 0.088 among screen-positive patients in sites with versus without a health coach. Power analysis assumptions and other details are in Additional file 4.

#### Economic evaluation

Aim 2 will conduct PLCEA to measure the cost of each implementation strategy in increasing reach, fidelity, and engagement.

To operationalize PLCEA, we will measure the following ratio:

$$\frac{\textrm{Incremental}\ \textrm{Population}\ \textrm{level}\ \textrm{Costs}}{\textrm{Incremental}\ \textrm{Population}\ \textrm{level}\ \textrm{effectiveness}}$$

where incremental population-level costs are the difference in costs between population in sites targeted by each implementation strategy. Population-level costs include implementation costs, direct intervention costs and indirect healthcare costs. While the primary outcome analyses above test specific hypotheses regarding the main effects followed by exploratory tests of interactions, the PLCEA will estimate the difference in the outcomes of reach, fidelity and engagement (see Outcome Measures) between respective populations targeted by one of the enhanced implementation strategies and the standard strategy. This produces information about incremental costs of each implementation approach while accounting for potential multiplicative effects on implementation costs [104]. Analyses will be performed from the perspective of a healthcare system and payer.

##### Implementation Costs

Implementation costs, which are collected for all trial arms, include the monetary value of time and resources incurred by the healthcare system needed to roll-out the digital therapeutics. We use an activity-based approach to calculate the opportunity cost of time devoted to activities related to implementation [105]. This approach uses microcosting, applying unit costs to the amount of time spent by multiplying the number of hours devoted to activities by the estimated wage rate of an implementation participant. Wage rates will be ascertained from Bureau of Labor Statistics data that captures the average wage for an implementation participant’s occupation in the geographical area of employment. Implementation costs also include the direct cost of implementation resources (e.g., printing cost of materials to support adoption). We will sum costs associated with activities and resources needed to execute all implementation strategies. Details about cost data collection are in Additional file 5.

##### Operating Costs

Operating costs are dollars spent on regular operation of the digital therapeutics incurred by the healthcare system and patients. We include both direct operating and indirect overhead costs for completeness. Direct costs include digital therapeutic licensing fees. Indirect cost expenditures include infrastructure/technical resources and staff support such as information technology, office space, and capital equipment. We will estimate infrastructure costs from public data sources following methods used in prior implementation cost studies [106].

##### Direct Intervention Costs

Intervention costs include encounters with clinicians related to the initiation and continued use of reSET and reSET-O. We will identify patient visits with app prescribers and health coaches during the 12-week prescription period, using EHR data. We will count encounters on the day of and in 12-weeks after patients activate their prescription.

Using patient-level encounter records, we will calculate intervention costs using KPWA’s internal accounting that measures actual production costs incurred by the healthcare system in providing care to members. This model allocates costs to all encounters based on a general ledger for all services. Cost values include a direct care component (e.g., nurse salaries) or an overhead component (e.g., facilities). Costs excluded from allocation include those not directly related to delivering health services (e.g., insurance) and patient out-of-pocket costs.

##### Other Indirect Healthcare Costs

Digital therapeutics usage may impact patients’ use of services other than SUD care. Thus, we will sum costs for all outpatient encounters (including primary care, specialty mental health, specialty medical, ancillary, acute care) and dispensed medications expected in this population.

### Qualitative Formative Evaluation of Implementation

#### Evaluation goals and methods

Formative evaluation is used to continuously identify and document modifications, adaptations, and implementation determinants (i.e., barriers and facilitators) as they pertain to each DSF construct. Evaluators communicate salient findings with the implementation team [70]. Evaluators also monitor for contamination across implementation strategy conditions and observe how implementation outcomes change in correspondence to changes in DSF constructs. The Framework for Reporting Adaptations and Modifications to Evidence-based Implementation Strategies (FRAME-IS) guides tracking of modifications, adaptations, processes of and reasons for change [107, 108].

Formative evaluation analyses follow a qualitative rapid assessment process [109, 110]. Using templated forms (Additional file 6), evaluators take field notes at implementation meetings and review secondary data sources (e.g., meeting notes, communications, and implementation documents). Analysis procedures consist of structured data reduction to categorize and summarize fieldnotes and secondary data sources according to predetermined domains [90, 107, 111], while also capturing emergent findings (e.g., decision points). Summary data are entered into a strategy-by-domain matrix to systematically extract themes, detect trends, and answer main evaluation questions (Additional file 7). Synthetized findings and recommended adaptations are regularly presented to the study team and healthcare system partners, who decide by consensus whether to execute adaptations.

#### Sustainment

Following the end of the active implementation period, funding and support for implementation strategies by the research grant is scaled back to study sustainment and institutionalization of the digital therapeutics. The grant will provide additional prescriptions after the active implementation period ends to allow time for the health system to decide whether to continue offering reSET and reSET-O, and to allow for the continued measurement of reach and fidelity and qualitative measures of sustainment.

#### Trial Status

No outcome data have been analyzed yet; the last site completes active implementation on 2/10/2023.

## Discussion

This trial is designed to overcome several limitations of prior research on digital treatments for SUD. Real-world implementation studies in health systems may provide more actionable information to decision makers such as health system leaders than traditional effectiveness trials [81, 84]. For instance, other trials may be conducted within selected patient populations who consent to participate in studies of treatment for SUD, who may not be representative of typical patients. Moreover, other trials may rely on researchers to deliver digital treatments whose workloads and primary responsibilities are very different from those who are delivering clinical care.

This study fills a gap in implementation science, where evidence is needed on the comparative effectiveness and cost-effectiveness of different implementation strategies on improving care. Findings regarding the population-level cost effectiveness of implementation strategies will further provide information to decisions makers about the financial implications of this study’s strategies. For instance, while an implementation strategy may result in greater effectiveness, it may not necessarily be more cost-effective. Other aspects of this study, such as the workflows created and the insights into sustainment ascertained through qualitative research methods, will help create a roadmap for other health care organizations wishing to care more effectively for SUDs.

### Strengths and Limitations

Significant strengths of this trial include that 1) the health system has high universal screening rates for substance use that can assist with comprehensively identifying the population targeted (i.e., the reach denominator); 2) the study has access to a diverse set of data enabling the detailed analysis of clinical and economic outcomes, and 3); the study is built on principles of preserving real-world conditions. However, the study design also presents limitations. Notably, the study is being conducted after the COVID pandemic began; in the context of significant healthcare system staffing shortages, fewer clinics were recruited, and the intervention is delivered in clinics with reduced capacity. The substance use and abstinence measures collected from the EHR will be available only for an estimated ~70% of patients because of reliance on follow-up screening data. Findings may not generalize into systems that primarily serve uninsured populations.

## Conclusion

This trial seeks to understand how to best engage primary care clinicians and patients to increase the reach and fidelity of digital therapeutics for SUD. As opposed to providing data on clinical and cost outcomes associated with a digital therapeutic, this study will provide health system leaders with data on the outcomes of using specific implementation strategies.

## Supplementary Information

**Additional file 1. **Description of primary care team structure and availability of substance use disorder care at the participating health system*.*

**Additional file 2.** Identification and Selection of the Digital Therapeutic.

**Additional file 3.** Specification and reporting of the DIGITS Trial implementation strategies.

**Additional file 4.** Statistical power analysis details. Additional Information about Implementation Costs Data Collection.

**Additional file 5.** Additional Information about Implementation Costs Data Collection.

**Additional file 6.** Formative Evaluation Field Notes Template. DIGITS Trial Meeting Field Notes – Active Implementation.

**Additional file 7.** Formative evaluation questions.

## Data Availability

Economic and formative data collection materials are provided as Additional Files. Complete details on the operationalization of measures using electronic health data are outlined in the Statistical Analysis Plan (available from authors). The trial’s Data Safety and Monitoring Plan and protocol amendments are available from the first author upon reasonable request. Additional study materials and data are available from the first author upon reasonable request. Individual-level data will not be released to avoid risk of participant re-identification.

## Abbreviations

DIGITS Trial: The Digital Therapeutics for Opioids and Other Substance Use Disorders Trial
DSF: Dynamic sustainability framework
EHR: Electronic health records
FDA: United States Food and Drug Administration
FRAME-IS: Framework for Reporting Adaptations and Modifications to Evidence-based Implementation Strategies (FRAME-IS)
HIPAA: Health Insurance Portability and Accountability Act
HIT-ACE: Health Information Technologies-Academic and Commercial Evaluation
KPWA: Kaiser Permanente Washington
MA: Medical assistant
SUD: Substance use disorder
OUD: opioid use disorder
PCP: Primary care provider
PDSA: Plan-Do-Study-Act
PLCEA: Population-level cost effectiveness analysis
RCT: Randomized controlled trial
