# Supplementary material for: Study protocol for a factorial-randomized controlled trial evaluating the implementation, costs, effectiveness, and sustainment of digital therapeutics for substance use disorder in primary care (DIGITS Trial)
Source: Implement Sci. 2023 Feb 1;18:3. doi: 10.1186/s13012-022-01258-9 (PMC9893639; doi:10.1186/s13012-022-01258-9)
Supplement: Supplementary file 1 — Additional file 1. Description of primary care team structure and availability of substance use disorder care at the participating health system. [file 13012_2022_1258_MOESM1_ESM.docx]

### Additional file 1: Description of primary care team structure and availability of substance use disorder care at the participating health system

In primary care, teams of MAs, primary care providers (PCPs), nurses, and integrated mental health specialists who are Licensed Independent Clinical Social Workers collaborate to provide healthcare to the patient. Integrated mental health services include population-based screening, symptom assessment, and care for alcohol, cannabis, other drug use, and depression [1]. 80-90% of primary care patients are screened annually for these conditions. Many clinics have PCPs who are buprenorphine medication prescribers, which is a first-line treatment for opioid use disorders often provided in primary care. The healthcare system operates an Addiction Recovery Service, which is available in certain catchment areas, but most clinics access specialist addiction treatment services through an externally contracted care network.

**Reference**

1. Glass JE, Bobb JF, Lee AK, Richards JE, Lapham GT, Ludman E, et al. Study protocol: A cluster-randomized trial implementing Sustained Patient-centered Alcohol-related Care (SPARC trial). Implement Sci. 2018;13:108.
