# Supplementary material for: Study protocol for a factorial-randomized controlled trial evaluating the implementation, costs, effectiveness, and sustainment of digital therapeutics for substance use disorder in primary care (DIGITS Trial)
Source: Implement Sci. 2023 Feb 1;18:3. doi: 10.1186/s13012-022-01258-9 (PMC9893639; doi:10.1186/s13012-022-01258-9)
Supplement: Supplementary file 2 — Additional file 2. Identification and Selection of the Digital Therapeutic. [file 13012_2022_1258_MOESM2_ESM.docx]

### Additional file 2: Identification and Selection of the Digital Therapeutic

Healthcare leaders at the study site had experience implementing a digital therapeutic for depression and anxiety in primary care and expressed interest in partnering to study how to use digital therapeutics to address substance use disorders. To determine the best product for testing the principles of promoting the implementation and sustainment of a new digital therapeutic, the research team partnered with these leaders to apply the first two phases of the Health Information Technologies-Academic and Commercial Evaluation (HIT-ACE) methodology [1]. First, the PI catalogued commercialized digital products for substance use disorders, identifying their capabilities (e.g., clinical functions) and characteristics (e.g., evidence). Next, researchers met with product vendors to gain additional information and reported back to healthcare system partners. Vendors then met with healthcare system leaders and local design experts. Finally, the PI visited offices of several vendors to build relationships, and vendors visited research and healthcare offices to present to additional stakeholders.

reSET and reSET-O were chosen for this study for multiple reasons. First, they are authorized by the FDA and can be prescribed, potentially providing legitimacy in a medical setting and a pathway for insurance reimbursement. Second, strong inferential evidence from studies in specialty care supported reSET and reSET-O. Third, the Therapeutic Educational System, which is the research product that reSET and reSET-O are based on, has been shown to have high acceptability in diverse populations, including Black/African American, Hispanic/Latino, and American Indian/Alaskan Native research participants [2,3]. Fourth, reSET and reSET-O had important features for primary care: a well-designed patient interface, a smartphone app, and a web-based interface to monitor patient engagement with the app. Fifth, reSET-O was designed to be used in conjunction with buprenorphine medication treatment. The combination of the reSET and reSET-O products allowed an implementation study relevant to many drug use disorders, rather than studying the implementation of treatments for specific disorders in separate studies (e.g., opioids, cannabis, stimulants). Sixth, the intervention arms in these trials were designed to reduce clinician time by 50-80% by providing most psychosocial interventions electronically. Such an approach where clinician time to treat substance use disorder could be reduced in primary care would be advantageous. Seventh, in a multisite trial, effects were even stronger among patients who were actively using substances when they started treatment [4], which is relevant in primary care in part because patients can be opportunistically offered interventions based on their current substance use reported on population-based screenings.

**References**

1. Lyon AR, Lewis CC, Melvin A, Boyd M, Nicodimos S, Liu FF, et al. Health Information Technologies-Academic and Commercial Evaluation (HIT-ACE) methodology: description and application to clinical feedback systems. Implement Sci. 2016;11:128.

2. Campbell AN, Turrigiano E, Moore M, Miele GM, Rieckmann T, Hu MC, et al. Acceptability of a web-based community reinforcement approach for substance use disorders with treatment-seeking American Indians/Alaska Natives. Community Ment Health J. 2015;51:393–403.

3. Campbell ANC, Montgomery L, Sanchez K, Pavlicova M, Hu M, Newville H, et al. Racial/ethnic subgroup differences in outcomes and acceptability of an Internet-delivered intervention for substance use disorders. J Ethn Subst Abuse. 2017;1–19.

4. Campbell AN, Nunes EV, Matthews AG, Stitzer M, Miele GM, Polsky D, et al. Internet-delivered treatment for substance abuse: a multisite randomized controlled trial. Am J Psychiatry. 2014;171:683–90.
