# Supplementary material for: Study protocol for a factorial-randomized controlled trial evaluating the implementation, costs, effectiveness, and sustainment of digital therapeutics for substance use disorder in primary care (DIGITS Trial)
Source: Implement Sci. 2023 Feb 1;18:3. doi: 10.1186/s13012-022-01258-9 (PMC9893639; doi:10.1186/s13012-022-01258-9)
Supplement: Supplementary file 3 — Additional file 3. Specification and reporting of the DIGITS Trial implementation strategies. [file 13012_2022_1258_MOESM3_ESM.docx]

### Additional file 3: Specification and reporting of the DIGITS Trial implementation strategies

Additional file 3 Tables A, B, and C contains the specification and reporting of the DIGITS Trial’s standard implementation, practice facilitation, and health coaching implementation strategies, respectively. These tables follow reporting guidelines outlined by Proctor and colleagues [1], which recommend that researcher name, define, and specify the implementation strategy. Specifying the implementation strategy involves identifying the actor, action, action target, temporality, dose, implementation outcomes affected, and justification for the strategy. Hypotheses in relation to primary implementation outcomes, and justification for each strategy exists in the main text.

| **Additional file 3 Table A.** Specification and reporting of the DIGITS Trial standard implementation strategy | |
| --- | --- |
| Description and Actor: The healthcare system encourages integrated mental health specialists and other primary care team members to offer digital therapeutics to patients | |
| Actions | Corresponding Conceptual Targets |
| 1. Conduct educational meetings on background, use, and provision of the digital therapeutic, use of new electronic health record (EHR) tools    2. Develop and distribute educational materials in the form of an implementation toolkit: detailed instructions on how to provide the digital therapeutic, patient education pamphlet, and vendor materials (video, access to the digital therapeutic)    3. Develop and implement tools for quality monitoring and intervention delivery using EHR tools: digital therapeutic order set, documentation templates, population management workbench    4. Provide clinical supervision focusing on the digital therapeutic in standing or ad-hoc meetings    5. Obtain policy approvals to deliver motivational incentives that are packaged with the digital therapeutic, authorize social workers and nurses to prescribe with physician consultation    6. Draw contract for the digital therapeutic to facilitate agreement between national and regional health system offices and vendor re: data privacy, intellectual property, and liability    7.  Select pilot clinics to stage implementation scale-up and encourage ongoing small cyclical tests of change before implementing changes system-wide | 1 & 2: Increase knowledge about the apps and processes for delivering them  3: Document and encourage processes for producing patient and implementation outcomes, ensure fidelity  4: Encourage fidelity, skill building, and troubleshooting barriers  5:  Fulfill practice setting policy requirements  6: Formally agree on & clarify responsibilities  7: Maximize fit between the practice setting, clinician, intervention, and patient |
| Temporality and Dose: Implementation materials are prepared in advance for initial launch trainings that ensue and are updated as needed on a regular basis. Trainings to prescribe reSET and reSET-O last 2-3 hours for integrated mental health specialists and approximately 1 hour for primary care providers and nurses. Regional support and 1:1 supervision are provided as needed. Contracting and policy approvals are completed before implementation activities ensue. Implementation is staged with 6 months of piloting prior to system-wide rollout. Units of analysis are described in the manuscript text. | |

| **Additional file 3 Table B.** Specification and reporting of the DIGITS Trial practice facilitation implementation strategy | |
| --- | --- |
| Description and Actor: Trained practice facilitators from the research team help overcome workflow challenges by supporting clinicians in tailoring implementation to their local context | |
| Actions | Corresponding conceptual targets |
| Practice facilitators work directly with clinics to guide tailoring implementation to their local context needs.  Practice facilitators deliver four interventions in the context of a supportive relationship:  1. Bolster education: Help reSET and reSET-O prescribers learn how to market the digital therapeutics to patients and primary care teams and garner support for digital therapeutic use. Provide or connect individuals to technical assistance to support the use of the digital therapeutics.  2. Audit and provide feedback: Share progress on measurable performance goals of reach and fidelity for self-assessment and individual performance ranking in comparison to other anonymized clinics to prompt change in practice.  3. Plan-Do-Study-Act (PDSA) cycles: Support the clinic implementation team in designing small tests of change to increase intervention reach and fidelity. Study through review of audit and feedback data, problem solve, and adjust activities for continuous improvement and increased workflow efficiencies.  4. Engage others in change: Invite additional implementation stakeholders (e.g. clinic leadership, primary care providers, care team members) to participate in problem-solving and PDSA cycles. | 1. Bolster enthusiasm about the intervention and create clinic-wide demand  2. Clarify measurable goals to improve individual performance  3. Maximize fit by generating continuous and reciprocal feedback on the intervention  4. Engage multiple care team members and stakeholders and champions to support the local implementation effort |
| Temporality and Dose: Practice facilitators provide 1 meeting with the clinic’s digital therapeutic prescriber after randomization, 1 meeting with clinic leadership in Month 1, 1 clinic implementation team kickoff meeting in Month 1, One monthly facilitation visit during active implementation (12 in total including kickoff meeting), ad hoc support during implementation via email, phone or videoconferencing. Overall, the facilitators provides up to five hours of time per clinic per month. Units of analysis are described in the manuscript text. | |

| **Additional file 3 Table C.** Specification and reporting of the DIGITS Trial health coaching implementation strategy | |
| --- | --- |
| Description and Actor: A centralized Medical Assistant from the healthcare system supports patients in engaging in the digital therapeutic while minimizing burden on primary care clinics | |
| Actions | Corresponding conceptual targets |
| 1. Phone outreach to patients who might benefit from the app  2. Monitor patient engagement with the clinician dashboard and reach out to patients not engaged  3. Reinforce digital therapeutic use over 12 weeks in health coaching calls and messages through the electronic health record-based patient portal  4. Encourage patient practice of skills learned from digital therapeutic content  5. Monitor and encourage patient follow-up with their care teams  The health coach is supported by:  6. Training to increase knowledge about health coaching  7. Obtaining certification in health coaching  8. Technical support from a research interventionist to execute a structured protocol  9. Clinical supervision from the healthcare system | 1 & 2: Activate patients in care with digital therapeutic  3 & 4: Inform/educate patients by supporting them in skill development  5: Promote collaboration between patients and healthcare professionals  6: Increase knowledge about health coaching and the process for delivering it  7: Maintain professional standards for conducting health coaching  8: Promote standardization and fidelity  9: Encourage skill building, troubleshooting barriers, and satisfying regulatory requirements |
| Temporarily and Dose: The health coach spaces out contacts with patients approximately weekly during the 12-week prescription. Patients receive 4 telephone support sessions (approximately 30 mins), followed by 5 electronic messages. Project-specific training is conducted over approximately four hours, and 1-hr technical support from a research interventionist is held approximately weekly. Units of analysis are described in the manuscript text. | |

**Reference**

1. Proctor EK, Powell BJ, McMillen JC. Implementation strategies: recommendations for specifying and reporting. Implement Sci. 2013;8:139.
