# Supplementary material for: Study protocol for a factorial-randomized controlled trial evaluating the implementation, costs, effectiveness, and sustainment of digital therapeutics for substance use disorder in primary care (DIGITS Trial)
Source: Implement Sci. 2023 Feb 1;18:3. doi: 10.1186/s13012-022-01258-9 (PMC9893639; doi:10.1186/s13012-022-01258-9)
Supplement: Supplementary file 4 — Additional file 4. Statistical power analysis details. Additional Information about Implementation Costs Data Collection. [file 13012_2022_1258_MOESM4_ESM.docx]

### Additional file 4: Statistical power analysis details

Minimal detectable differences for fixed 80% power were estimated based on 27 clinics (number of clinics available during the study pilot phase) and a two-sided type 1 error rate of 0.025 for the two primary outcomes of reach and fidelity (to control the familywise type 1 error at 0.05). We will have >0.80 power to detect an increase of 2 percentage points in clinic-level reach among screen-positive patients in clinics with versus without practice facilitation, assuming a standard deviation (SD) across clinics of the percentage reached of 1.6 (based on preliminary data on digital depression treatment implemented previously). This corresponds to a 12 percentage point increase among those eligible for reSET or reSET-O (assuming that 16.6% of screen-positive patients are eligible based on baseline data). We estimated that we will have >0.80 power to detect an increase in the clinic-level mean number of weeks fidelity of 0.088 among screen-positive patients in clinics with versus without a health coach, assuming a SD across clinics of 0.07. To provide context, this difference of 0.088 weeks fidelity among screen-positive patients corresponds to an increase in the mean number of weeks fidelity among those reached of 4.5 (from 4 to 8.5 weeks), if reach among clinics with a health coach increases by 25% (e.g., from 1.3% to 1.7% among screen-positive patients [8% to 10% among those eligible]).
