# Supplementary material for: Study protocol for a factorial-randomized controlled trial evaluating the implementation, costs, effectiveness, and sustainment of digital therapeutics for substance use disorder in primary care (DIGITS Trial)
Source: Implement Sci. 2023 Feb 1;18:3. doi: 10.1186/s13012-022-01258-9 (PMC9893639; doi:10.1186/s13012-022-01258-9)
Supplement: Supplementary file 5 — Additional file 5. Additional Information about Implementation Costs Data Collection. [file 13012_2022_1258_MOESM5_ESM.docx]

### Additional file 5: Additional Information about Implementation Costs Data Collection

Implementation costs data collection in this study uses microcosting, applying unit costs to the amount of time spent by multiplying the number of hours devoted to activities by the estimated wage rate of an implementation participant. Implementation costs also include the direct cost of implementation resources (e.g., printing cost of materials to support adoption). Data collection tools used in this study to collect economic costs were adapted from prior research studies [1,2].

Data collection related to implementation activities and implementation participant wages

Time spent on implementation activities are collected for all participants involved in implementation including healthcare system leaders, practice facilitators, health coaches, clinicians who prescribe reSET and reSET-O, and other personnel involved. There are four data collection components:

1. Study team members complete time tracking spreadsheets on monthly basis from start-up through active implementation phases of the project. A designated study team member sends these spreadsheets via email, tracks responses, and sends reminders to ensure completion.

2. Healthcare system leadership and staff who assist with the implementation complete time tracking spreadsheets on a biweekly or monthly basis from start-up through active implementation phases of the project. A designated study team member sends these spreadsheets via email and reminds recipients to complete the spreadsheets at recurring implementation operations meetings to ensure completion.

3. Primary care members who are reSET and reSET-O prescribers estimate time spent offering reSET and reSET-O in the previous week by completing an email form. To reduce reporting burden, these individuals receive only one request during the entire active implementation phase. A data collection schedule spaces the emails across study weeks. A study team member uses a random number generator to select the email recipient during any given week. A study team member sends the initial email and up to two reminders to ensure completion. Because individuals receive only one request during the implementation phase, time devoted to implementation activities by primary care members will not be available for each week. We will extrapolate time estimates for these weeks using a linear regression and applying the method of recycled predictions.

4. To reduce documentation burden and avoid duplication, participants completing instruments 1-3 above are instructed to omit time spent in implementation meetings organized by the study team. Instead, a study team member reviews meeting notes, agendas, and calendar invites to identify time that participants spend in these meetings. Meeting information is documented in a spreadsheet (e.g., title, description, duration, attendees) for later analysis.

Figures A through D below contain a screenshot of the primary data collection tools used to collect of the economic analysis during the implementation phase (earlier versions were used during the piloting phases). To monetize time on implementation activities, we will multiply hours of participation by opportunity cost of time, measured as participant’s estimated hourly wage rate. We will inflation-adjust all costs to 2022 constant dollars using the Personal Health Care Index, as suggested in prior research [3]. We will use aggregate data from the Bureau of Labor Statistics on average wages and employee benefit costs by area and occupation [4].

Direct costs of materials and resources

Implementation costs also include the direct costs of materials and resources required to support implementation of reSET and reSET-O. Examples include the printing cost of patient pamphlets, guides, health coach training and certification costs, and training materials. These direct costs will be ascertained from study budget records.

**Figure A.** Screenshot of the time tracker spreadsheet completed by individual study team members during the active implementation phase. Earlier versions were used during piloting phases.


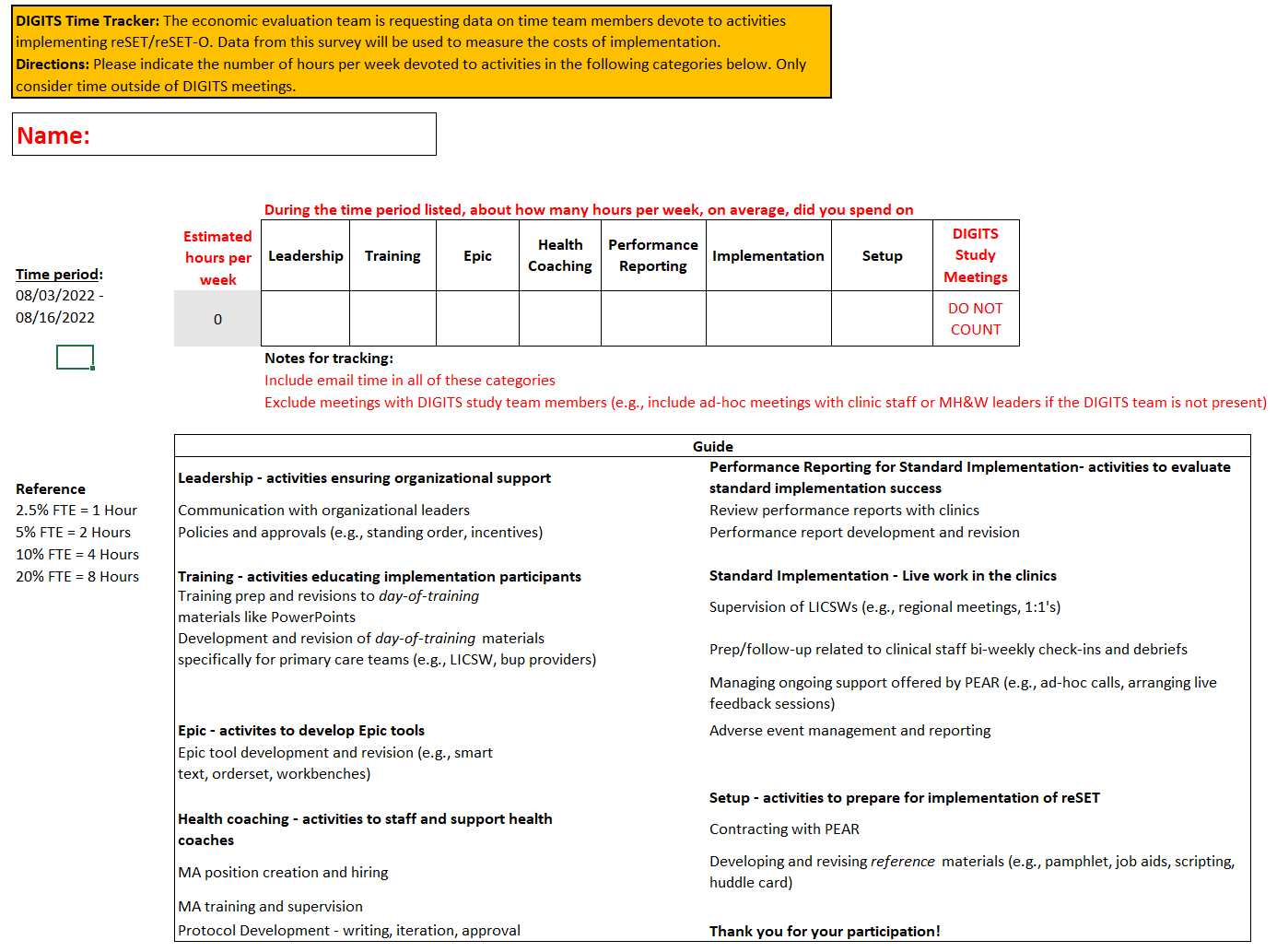


**Figure B**. Screenshot of the time tracker spreadsheet completed by healthcare system partners who served on the implementation team


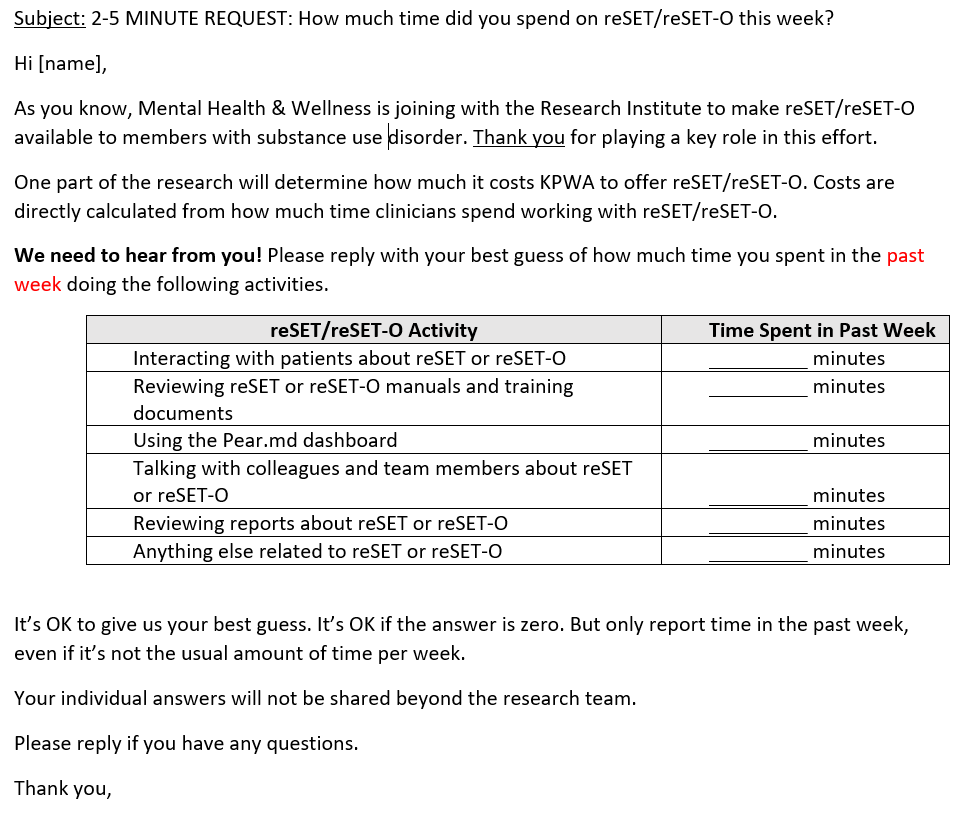


**Figure C.** Screenshot of the email template used to collect time data from reSET and reSET-O prescribers


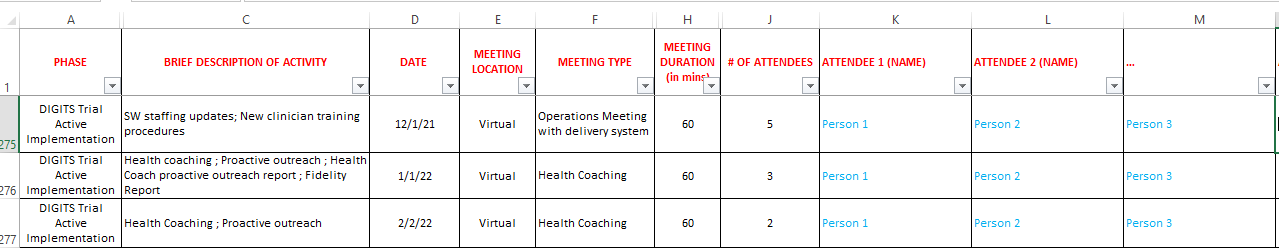


**Figure D.** Screenshot of the spreadsheet fields collected by a study team member about time spent during implementation meetings. Data shown in the rows of this screenshot are fake data.

**References**

1. Fortney JC, Pyne JM, Burgess JF. Population-level cost-effectiveness of implementing evidence-based practices into routine care. Health Serv Res. 2014;49:1832–51.

2. Liu CF, Rubenstein LV, Kirchner JE, Fortney JC, Perkins MW, Ober SK, et al. Organizational cost of quality improvement for depression care. Health Serv Res. 2009;44:225–44.

3. Dunn A, Grosse SD, Zuvekas SH. Adjusting health expenditures for inflation: A review of measures for health services research in the United States. Health Serv Res. 2018;53:175–96.

4. US Bureau of Labor Statistics. Occupational Employment and Wage Statistics [Internet]. Available from: https://www.bls.gov/oes/
