# Supplementary material for: Study protocol for a factorial-randomized controlled trial evaluating the implementation, costs, effectiveness, and sustainment of digital therapeutics for substance use disorder in primary care (DIGITS Trial)
Source: Implement Sci. 2023 Feb 1;18:3. doi: 10.1186/s13012-022-01258-9 (PMC9893639; doi:10.1186/s13012-022-01258-9)
Supplement: Supplementary file 6 — Additional file 6. Formative Evaluation Field Notes Template. DIGITS Trial Meeting Field Notes – Active Implementation. [file 13012_2022_1258_MOESM6_ESM.docx]

### Additional file 6: Formative Evaluation Field Notes Template

**DIGITS Trial Meeting Field Notes – Active Implementation**

**This field notes template** is completed by research team member(s) attending stakeholder meetings**.** If ≥2 team members attend, one is identified as the lead note taker and the other team member(s) amend the document as needed. Notes may be taken during or after the meeting.

**Meeting date** **and** **time**:

**Meeting name**:

**In-person attendees**:

**Remote attendees**:

**Meeting location**:

**Fieldnotes author:**

Meeting Agenda Items/Summary paragraph*

*Please indicate if secondary data such as meeting minutes were used to complete this template in place of or to augment direct observation data.

**Observations were conducted regarding:**

Barriers

Facilitators

Modifications

☐ Implementation Strategies

*Mark the Implementation Strategies box above whenever observations regarding implementation strategies were included. Use the checkboxes below to indicate the implementation strategy actions, aspects of temporarily, and/or dose (Proctor et al., 2013) when observed and recorded in these field notes. Aspects of the strategy corresponding to the checkboxes may help when analyzing the fieldnotes (e.g., when forming an initial list of codes, determining whether/how the strategy is being applied with fidelity).*

*Write your detailed notes about actions, temporality, and/or dose in the space under the checkboxes or in the free-form notes/reflections section, under Implementation Strategies.*

*“Actions” refer to the specific actions, steps, or processes that need to be enacted. “Temporality” refers to when things happen to enact the strategy. “Dose” refers to the “amount” of strategy used (such how long something took or how much time was devoted.)*

**Standard Implementation**

***Actions: Standard Implementation***

1. Conduct educational meetings

2. Develop and distribute educational materials

3. Develop and implement tools for quality monitoring

4. Provide clinical supervision

5. Obtain policy approvals

6. Contract for the digital therapeutic

7. Select pilot clinics to stage implementation scale-up and encourage ongoing small

cyclical tests of change

***Temporality: Standard Implementation***

Materials are prepared before implementation launch and are modified as needed

Initial trainings occur at implementation launch and ongoing as needed (LICSW

group supervision calls, 1:1 supervision as needed)

Contracting and policy approvals are completed as needed and time is recorded

Implementation is staged with 6 months of piloting prior to system-wide rollout

***Dose: Standard Implementation***

Initial 3-hour training

Ad hoc education at LICSW group supervision/other calls

**Practice Facilitation**

***Actions:*** ***Practice Facilitation***

Education: how to market to patients and primary care teams

Audit and provide feedback: measurable performance goals of reach and fidelity for self-

assessment and individual performance ranking in comparison to others

Plan-Do-Study-Act cycles: review audit and feedback data, create improvement plan, study

and adjust activities for continuous improvement

Engage others in change: inform and influence additional implementation stakeholders

***Temporality: Practice Facilitation***

12-month intervention; monthly planned visits by Practice Facilitator, plus ad hoc meetings

Email feedback report to clinicians every month

***Dose: Practice Facilitation***

Up to five hours facilitator time per clinic per month, including planned one-hour visits and ad-

hoc meetings

**Health Coaching**

***Actions: Health Coaching***

Monitor the vendor-supplied population management dashboard and reach out to patients not

engaged

Reinforce digital therapeutic use

Encourage practice of skills learned from digital therapeutic content

Monitor and encourage patient follow-up with their care teams

***Temporality: Health Coaching***

2 project-specific training and technical assistance trainings

Booster training

12 weekly telephone support sessions

***Dose: Health Coaching***

Project-specific training and technical assistance training: four-hour sessions

Booster training: 2-hour sessions

Weekly telephone or secure message support sessions:

Week 1: Welcome call

Week 2: Phone Outreach

Week 3: Phone Outreach

Week 4: Phone Outreach

Week 5: Secure Message Outreach (Note: patients who are not active on kp.org will continue to

receive phone outreach)

Week 7: Secure message if pt. using the app or phone outreach to reengage (or if not active on

kp.org)

Week 9: Secure message if pt. using the app or phone outreach to reengage (or if not active on

kp.org)

Week 11: Secure message if pt. using the app or phone outreach to reengage (or if not active on

kp.org)

Week 12: Final secure message or phone outreach

Quotes (record any patient stories or notable quotes here)

Vocabulary (record any new vocabulary learned here)

Other (e.g., decision points, surprises, noteworthy comments etc.)

**Free-form notes/reflections**

- **Implementation Strategies**:
- **Other Meeting Notes**:

**Glossary**

The glossary defines key implementation science terms found in this template and reflecting content to be captured in the field notes. See also the Additional file titled “Specification and reporting of the DIGITS Trial implementation strategies” attached to the DIGITS Trial protocol manuscript.

*Barrier*: Any factor that obstructs the capacity for the implementation of the intervention (e.g., insufficient staffing levels to carry out the intervention; competing priorities in the clinic) [1]

*Facilitator*: Any factor that enables the implementation of the intervention [1] (e.g., supportive leadership; programmer capacity to create new data reports)

*Modification*: Any changes made to interventions, whether deliberately and proactively (adaptation), or in reaction to unanticipated challenges that arise in a given session or context [2,3] (e.g., health system deviations from commercial offering of the prescription digital therapeutic; departures from protocols or training materials to tailor the intervention to local needs)

*Implementation Strategy*: Method or technique used to enhance the adoption, implementation, and sustainability of a clinical program or practice [4]

**References**

1. Bach-Mortensen AM, Lange BCL, Montgomery P. Barriers and facilitators to implementing evidence-based interventions among third sector organisations: a systematic review. Implementation Sci. 2018;13:103.

2. Miller CJ, Barnett ML, Baumann AA, Gutner CA, Wiltsey-Stirman S. The FRAME-IS: a framework for documenting modifications to implementation strategies in healthcare. Implement Sci. 2021;16:36.

3. Wiltsey Stirman S, Baumann AA, Miller CJ. The FRAME: an expanded framework for reporting adaptations and modifications to evidence-based interventions. Implementation Sci. 2019;14:58.

4. Proctor EK, Powell BJ, McMillen JC. Implementation strategies: recommendations for specifying and reporting. Implement Sci. 2013;8:139.
