# Supplementary material for: Study protocol for a factorial-randomized controlled trial evaluating the implementation, costs, effectiveness, and sustainment of digital therapeutics for substance use disorder in primary care (DIGITS Trial)
Source: Implement Sci. 2023 Feb 1;18:3. doi: 10.1186/s13012-022-01258-9 (PMC9893639; doi:10.1186/s13012-022-01258-9)
Supplement: Supplementary file 7 — Additional file 7. Formative evaluation questions. [file 13012_2022_1258_MOESM7_ESM.docx]

### Additional file 7: Formative evaluation questions

| **Additional file 7 Table A**. Formative evaluation questions and indicators | |
| --- | --- |
| Evaluation question | Indicators |
| - How are existing structures, systems and processes for the delivery of digital treatment (i.e., current practice) being used to support implementation?   - What are determinants of current practice? | - Leaders’, providers’, and practice facilitators perspectives on how current practice will support implementation - Background documents content that speaks to current practice that is relevant to implementation - Degree to which the implementation toolkit reflects/incorporates elements of current practice |
| - What were barriers and facilitators to adoption of the intervention? | - Perspectives/observations on barriers and facilitators to adoption |
| - What were barriers and facilitators that determined the intervention’s appropriateness to practice setting and ecological system under each trial condition? | - Perceptions of goodness of fit between the intervention, the setting where it is being implemented, and the broader context - Perspectives on barriers and facilitators to appropriateness |
| - What modifications occurred during implementation to maximize fit with the intervention, practice setting, and ecological system? | - Perspectives/observations on modifications to the intervention or implementation strategies - Domains from the FRAME-IS and the FRAME to systematically capture adaptations |
| - What was the experience of patients receiving and providers offering or supporting the intervention? | - Patient and provider satisfaction - Patient and provider perception of acceptability and quality of the intervention delivered or received |
| - To what extent was the intervention delivered as intended?   - What were barriers and facilitators implementation fidelity? | - Perspectives on determinants of fidelity - Consistency and congruence with core aspects of delivering the intervention as measured via observations |
| - To what extent are the practices and outcomes achieved by the clinics sustainable?   - What supports or hinders sustainability?   - What are lessons learned from implementation with implications for sustainability? | - Perspectives on sustainability of practices and outcomes achieved during the trial - Perspectives/observations of barriers and facilitators to sustainability - Identification of lessons learned for sustainability via interviews/observations - Extent to which practices have become embedded in clinics and the organization |
